# Reduced background autofluorescence for cell imaging using nanodiamonds and lanthanide chelates

**DOI:** 10.1038/s41598-018-22702-1

**Published:** 2018-03-14

**Authors:** Nicole M. Cordina, Nima Sayyadi, Lindsay M. Parker, Arun Everest-Dass, Louise J. Brown, Nicolle H. Packer

**Affiliations:** 1 ARC Centre of Excellence for Nanoscale BioPhotonics and Department of Molecular Sciences, Macquarie University, Sydney, NSW Australia; 2 Institute for Glycomics, Griffith University, Gold Coast, Queensland Australia

## Abstract

Bio-imaging is a key technique in tracking and monitoring important biological processes and fundamental biomolecular interactions, however the interference of background autofluorescence with targeted fluorophores is problematic for many bio-imaging applications. This study reports on two novel methods for reducing interference with cellular autofluorescence for bio-imaging. The first method uses fluorescent nanodiamonds (FNDs), containing nitrogen vacancy centers. FNDs emit at near-infrared wavelengths typically higher than most cellular autofluorescence; and when appropriately functionalized, can be used for background-free imaging of targeted biomolecules. The second method uses europium-chelating tags with long fluorescence lifetimes. These europium-chelating tags enhance background-free imaging due to the short fluorescent lifetimes of cellular autofluorescence. In this study, we used both methods to target E-selectin, a transmembrane glycoprotein that is activated by inflammation, to demonstrate background-free fluorescent staining in fixed endothelial cells. Our findings indicate that both FND and Europium based staining can improve fluorescent bio-imaging capabilities by reducing competition with cellular autofluorescence. 30 nm nanodiamonds coated with the E-selectin antibody was found to enable the most sensitive detective of E-selectin in inflamed cells, with a 40-fold increase in intensity detected.

## Introduction

Background autofluorescence is a major issue for the bio-imaging of cells and tissues. The natural emission of light by molecules that increase during enhanced cellular metabolism, such as flavins and NADH^1^, can interfere with the detection of fluorescent stains targeting specific cellular components. Conventional stains typically use antibodies conjugated to a fluorescent moiety such as an organic dye like fluorescein isothiocyanate (FITC). Differentiating between cellular autofluorescence and fluorescent staining from a dye is not always possible, particularly in cases where the expression of targeted molecules is low and the dye’s staining is weak. In such cases, only major changes in fluorescent intensity can be detected due to background autofluorescence.

In order to address the problem of autofluorescence in bio-imaging, alternative fluorescent species can be utilized. Nanoparticles such as fluorescent nanodiamonds (FNDs) have several properties that make them prime candidates for bio-imaging applications^2–5^. Defects in the sp^3^ carbon lattice core of nanodiamonds, such as nitrogen vacancy (NV) centers render these particles fluorescent when illuminated. Excitation at ~560 nm results in a broad emission spectrum with a maxima at ~700 nm. This FND-NV emission wavelength range is above the problematic background autofluorescence range of 450 to 670 nm^6^. It is therefore possible to use specific emission filters to limit the collection of the NV emission to longer wavelengths (700 nm), separate from any background autofluorescence.

**Figure 1:**
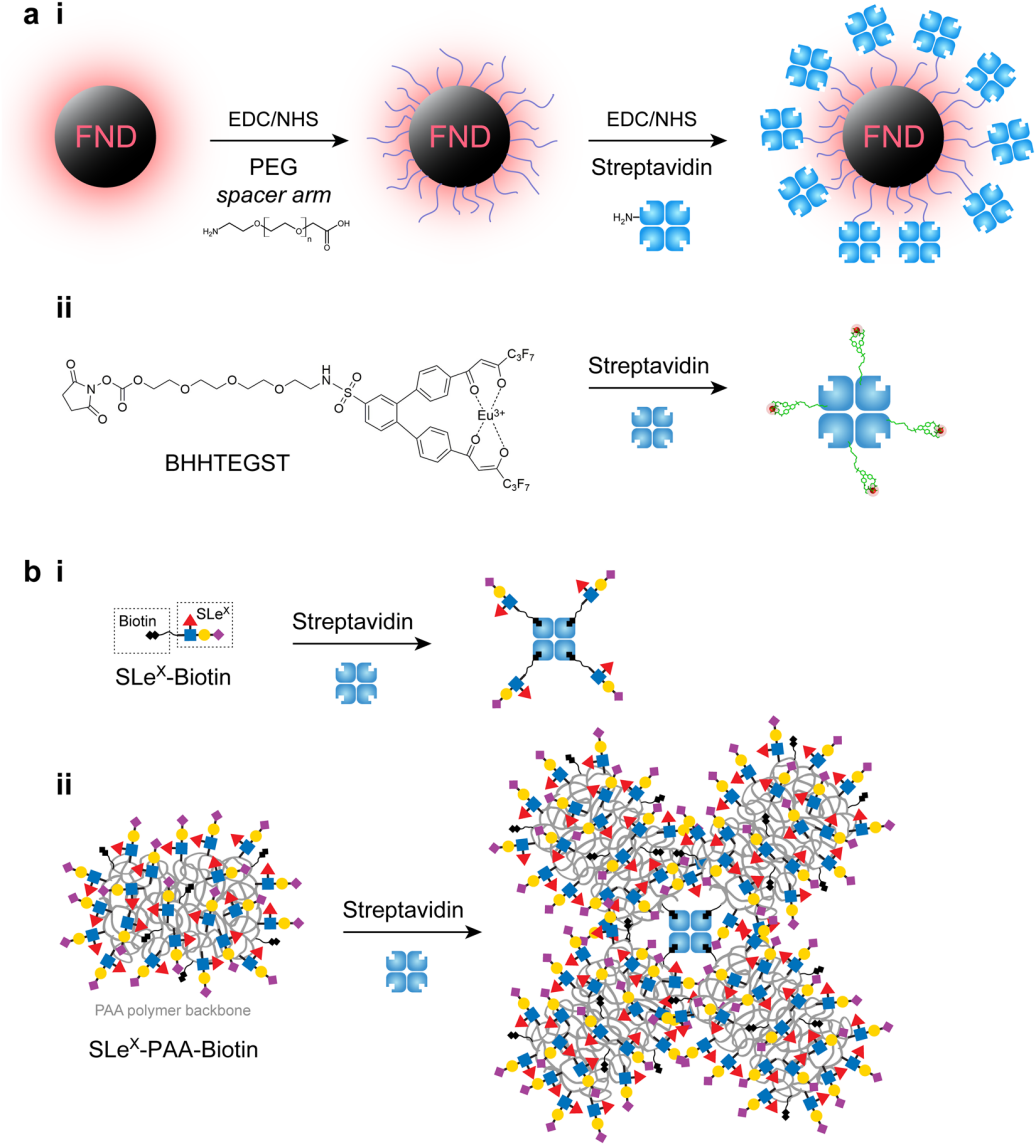
(**a**) The preparation of fluorescent probes for targeting E-selectin. (**ai**) FND-PEG-SA **-** After activation of carboxyl groups with EDC and NHS reagents, a PEG_22_ spacer arm was attached to the surface of 30 nm or 100 nm FNDs using the reagent carboxyl-PEG_22_-amine. The carboxyl groups at the terminus of the spacer arm were again activated with EDC/NHS for reaction with free amines on the surface of streptavidin (SA, blue). (**aii**) SA-BHHTEGST-Eu - The Europium ligand tag BHHTEGST^13^ was attached to streptavidin via lysine side chains. The size of chelate BHHTEGST was estimated at 3–4 nm, comparable to SA (~5 nm). (**b**) Biotinylated sialyl lewis X (SLe^X^) ligands used for targeting E-selectin. (**bi**) SLe^X^-biotin monovalent construct - Four of the monovalent SLe^X^ ligands (SLe^X^-biotin) are able to bind to each SA molecule, such that four SLe^X^ glycans can be displayed. (**bii**) SLe^X^-PAA-biotin multivalent construct - Up to four of the multivalent SLe^X^-PAA-biotin ligands, each with ~6 biotins and ~23 SLe^X^ ligands, can potentially bind with a SA molecule, which would result in the exposure of ~92 SLe^X^ glycans. Due to the flexibility of the PAA polymer backbone (grey), it is also feasible that multiple biotins from a single 30 kDa molecule of the multivalent ligand could occupy all four binding sites on SA.

When the NV point defects are positioned within the stable diamond core, the FNDs are extremely photostable. The FNDs are therefore not susceptible to photobleaching like conventional fluorophores^4^, nor do they exhibit photo-blinking such as observed with quantum dots^7^. These properties are the major advantage for the use of FNDs for bio-imaging, in particular for long-term tracking studies^8^. Additionally, the predominately sp^3^-carbon composition of FNDs makes them biologically inert, with many toxicology studies now demonstrating that FNDs are highly biocompatible^4,9^. While the core of the FND particles is stable, their surface is reactive. This allows for easy bio-functionalization and therefore specific targeting of an analyte within a biological environment^10^. FNDs with carboxylate functionalized surfaces are commercially available, or can be prepared easily by oxidizing their surface using techniques such as air oxidation or chemical refluxing in concentrated acids^3,10^. Amine-containing moieties can be covalently attached to the carboxylated surface of FNDs via simple carbodiimide crosslinking chemistry (Fig. 1a).

In order to target a biomolecule of interest with FNDs, the ligand of the target can be attached directly to the FND surface. Although it is possible to use direct chemical reactions for the attachment of specific ligands, a more universal system to attach FNDs to a range of ligands has clear advantages. The widely known streptavidin-biotin interaction can be conveniently utilized to facilitate such a strategy by covalently attaching the streptavidin (SA) directly to the surface of FNDs. The inclusion of a hydrophilic polyethylene glycol spacer arm (such as PEG_22_, Fig. 1ai) between the FND and SA increases the mobility of the SA molecule so that it retains selectivity and stability in aqueous solutions^11^. A biotinylated ligand for the chosen target can then be attached to the streptavidin-coated FND for targeting. This versatile ‘FND-PEG-SA’ scaffold can bind to any cellular target for which a biotinylated ligand can be generated.

Despite the ease with which FNDs can be labeled for targeted imaging, a number of size dependent properties of nanodiamonds must also be considered when designing a targeting experiment. FNDs are commercially available in a wide range of sizes (starting from ~10 to 20 nm, and going up to 500 nm or even larger). While FNDs of all sizes can typically be functionalized in the same manner using EDC/NHS chemistry, the ‘brightness’ of the FND depends upon the number of NV centers present within each particle. Larger FNDs (100 nm or greater in size) are naturally able to accommodate higher concentrations of these NV centers (more than 500 NV’s per particle), however smaller 30 nm FNDs typically contain 1 to 3 NV’s per particle^12^. Clearly, increased brightness is an advantage of larger diamonds, although there are naturally some advantages of using smaller FNDs for targeted bio-imaging applications. In particular, smaller FNDs conjugated to ligands are better able to bind to their targets without causing interference with the target’s biological function. For intracellular targets, the ability of FNDs to penetrate the cell membrane must also be considered.

While the long emission wavelengths of FNDs enable observation of NV emission free from the autofluorescent background, an alternative approach for eliminating background cellular autofluorescence without nanoparticles capitalizes on the property that autofluorescence has a short fluorescent lifetime. Luminescent compounds with long excited lifetimes enable time-gated image acquisition to be performed, eliminating cellular autofluorescence and thereby increasing the sensitivity of detection. Lanthanide metals, in the form of europium-chelating tags, are suitable for these time-gated imaging applications^13,14^. The streptavidin/biotinylated ligand system can be used with time-gated microscopy via a biocompatible europium chelating tag (such as BHHTEGST) covalently attached to the amines of the lysine residues in streptavidin (SA-BHHTEGST-Eu, Fig. 1aii)^13,14^. Although the toxicity of lanthanide-containing tags is not yet established, these tags are particularly helpful for *in vitro* and *ex vivo* imaging where the background autofluorescence level is high.

Though autofluorescence is observed in all cell types, it is particularly problematic for cells of the central nervous system due to the high levels of FAD/NADH expression that are known to contribute to this this background cellular fluorescence^15^. The fluorescent detection of physiological responses, such as neural inflammation, thus requires the use of background-free imaging strategies, such as those described here. In this work, the transmembrane glycoprotein E-selectin was targeted to demonstrate the application of both FND-PEG-SA and SA-BHHTEGST probes for the labeling of highly autofluorescent mouse brain endothelial cells. E-selectin is a key protein upregulated during the inflammatory response^16,17^. During the inflammatory response, leukocytes are recruited from blood vessels to the site of an injury in order to heal the damaged tissue. Leukocytes must first adhere to the blood vessel wall. This is achieved through the cell surface expression of the selectin family of cell adhesion molecules (CAMs), which include E-selectin, in response to cytokines released by injured tissue^18^. E-selectin recognizes the Sialyl Lewis carbohydrate motifs (SLe^X^, SLe^A^) found on glycoprotein and/or glycolipids on the leukocyte cell surface^19–21^.

E-selectin has previously been targeted using antibodies modified with the fluorescent tag Dylight-750^22^. Dylight tags, and other commercially available fluorophores attached to antibodies, are prone to rapid photobleaching; thus targeting E-selectin with non-bleaching tags is clearly advantageous because it enables the acquisition of multiple images without concern of signal loss, which is particularly important when imaging in a highly autofluorescent environment. Since SLe^X^ is a natural ligand of E-selectin, conjugating this glycan to nanodiamonds to target E-selectin is rational. Metallic nanoparticles, quantum dots, and polymeric nanoparticles functionalized with multivalent carbohydrates (glyconanoparticles) have been shown to bind strongly with other lectins^23^.

Comparison of the expression levels of E-selectin in rat brain endothelial cells during inflammation was therefore performed using a biotinylayed SLe^X^ ligand (Fig. 1bi) attached to SA-conjugated FNDs and SA-BHHTEGST chelated europium scaffolds (Fig. 1ai and ii, respectively). A multivalent form of the SLe^X^ ligand was also used for comparison, to see whether the presence of multiple SLe^X^ ligands per SA unit enhanced binding (Fig. 1bii). Inflammation was induced using the cytokine TNFα. The E-selectin antibody was also directly conjugated to FNDs for comparison of more conventional antibody approaches for nanoparticle targeting. The effectiveness of ligand targeting of E-selectin with FNDs as a fluorescent probe was compared to the time-gated detection using the long luminescent lifetime SA-BHHTEGST-Eu construct^13^. Our results demonstrated that both ligand conjugated FNDs and SA-BHHTEGST-Eu can enable the background-free detection of E-selectin within highly autofluorescent mouse brain endothelial cells. However, the utilization of the E-selectin antibody attached to 30 nm FNDs was found to offer the most sensitive detection of E-selectin in inflamed cells.

## Results

### Conjugation of europium chelating tag to streptavidin: SA-BHHTEGST

BHHTEGST was synthesized and purified as previously described^13^. High resolution mass spectrometry and NMR spectroscopy were used to verify the synthetic product, as previously reported^13^. BHHTEGST contains an *N*-hydroxysuccinimide ester that enables its attachment to streptavidin via the amino group of lysine residues. To increase the luminescent output and consequently the detection sensitivity of luminescent probes it is a common strategy to attach a maximum number of chelating tags onto a carrier molecule such as SA, thus a 30-fold molar excess of BHHTEGST to SA was used. At molar excesses above 30-fold, we observed a tendency for the conjugate to precipitate. UV-visible absorption spectroscopy was utilized to determine that the resultant molar ratio of BHHTEGST to SA was 18:1 (SA-BHHTEGST_18_)^13,14^.

### Preparation of streptavidin-coated 30 nm and 100 nm fluorescent nanodiamonds: FND-PEG-SA

30 nm and 100 nm FNDs were functionalized with a PEG_22_ spacer arm and streptavidin in a two-step reaction, as shown in Fig. 1ai. The particle size distributions and zeta potential values (charge) of the FNDs during the functionalization process was monitored using DLS (Table 1 and Supplementary Figure S1). Unmodified nanodiamonds with carboxylated surfaces (FND-COOH) were observed to have a negative zeta potential values less than −30 mV. These values indicated an overall negative surface charge and suggested good colloidal stability in solution (Table 1). Typically, for a colloidal suspension to remain in a suitably disperse state, a zeta potential value greater than |30 mV| is desired^24^.

**Table 1:** DLS size distributions (number intensity) and zeta potential measurements for 30 nm and 100 nm FND samples during biofunctionalisation with PEG_22_ and streptavidin (SA) are listed.

FND size	Sample	Size (nm)	Width (nm)	Zeta Potential ± SD (mV)
30 nm	FND-COOH	36.42	8.96	−40.4 ± 22.8
	FND-PEG_22_	52.55	17.62	−42.3 ± 9.3
	FND-PEG_22_-SA	59.14	17.85	−47.7 ± 9.4
100 nm	FND-COOH	128.4	30.1	−34.3 ± 7.0
	FND-PEG_22_	139.7	27.4	−36.0 ± 7.0
	FND-PEG_22_-SA	149.3	25.6	−39.8 ± 6.6

All measurements were performed on samples of 0.01 mg/mL in DDW. Polydispersity index values were <0.3 for all measurements indicating a monodispersed distribution of particles within the samples tested (*not shown*).

After modification with PEG, the zeta potentials decreased for both the 30 nm and 100 nm FNDs (Table 1). These observations were consistent with a change in the solvation of the FNDs expected to accompany the successful addition of the PEG spacer arm. After their final modification with streptavidin, a further decrease in zeta potential (to a more stable value) was observed. The changes in particle size distributions observed by DLS were consistent with the successful addition of the PEG and SA moieties to the FNDs. The size of the extended PEG_22_ spacer arm is 8–9 nm, thus a uniform coating of FNDs with PEG should result in a size increase of 16–18 nm, assuming an extended conformation occurs after the conjugation. This was indeed observed for the 30 nm FNDs, as a size increase of 16.1 nm was observed after PEGylation (Table 1). The 100 nm FNDs, on the other hand, increased in size by 11.3 nm upon PEGylation, suggestive of a slightly collapsed conformation of the PEG spacer arm after conjugation. SA is approximately 5 nm across its largest dimension (PDB 3WYP^25^), thus a size increase of ~10 nm would be expected after its conjugation if SA adopted an ‘exposed’ configuration as depicted in Fig. 1ai. This was observed for the 100 nm FND-PEG, which increased in size by 9.6 nm after the addition of SA. 30 nm FND-PEG was found, however to increase in size by 6.6 nm, suggesting that the SA was partially ‘absorbed’ within the PEG layer.

To further confirm the successful functionalization of the FND surfaces with PEG and SA, and to assess the uniformity of the coating, a co-localization experiment was performed where 100 nm sized FND-PEG –SA was incubated with biotinylated FITC (Supplementary Figure S2). Clusters of FNDs were observed with confocal microscopy after dual excitation at 538 nm (FND excitation, red) and 488 nm (FITC excitation, green). The complete co-localization of red FND-NV fluorescence and green FITC fluorescence suggested a uniform SA coating on the FND particles.

### Conjugation of Sialyl Lewis X to Fluorescent Nanodiamonds

In order to target E-selectin with either the FNDs or europium ligand tag, the natural carbohydrate ligand of E-selectin, Sialyl Lewis X (SLe^X^), was utilized. Two alternative forms of this molecule were biotinylated (Fig. 1b) for the subsequent non-covalent attachment to FND-PEG-SA and SA-BHHTEGST via the high affinity SA-biotin interaction; the monovalent species (SLe^X^-biotin, Fig. 1bi) and the multivalent (polyacrylamide) polymer with attached SLe^X^ (SLe^X^-PAA-biotin, Fig. 1bii). The amount of SLe^X^ glycan attached to the surface of 100 nm FND-PEG-SA was quantified using the fluorescent lectin MAL 1-FITC, which binds to the SLe^X^ motif (see Supplementary data Table S1). Both SLe^X^ ligand presentations (monovalent and multivalent) were found to be capable of binding similar amounts of the lectin (0.54 μg lectin per mg of FND-PEG-SA/SLe^X^-biotin, and 0.50 μg lectin per mg of FND-PEG-SA/SLe^X^-PAA-biotin), indicating that the number of accessible SLe^X^ moieties on the FNDs with both ligands was essentially the same.

### Imaging of SLeX binding to E-selectin using 30 nm Fluorescent Nanodiamonds

**Figure 2:**
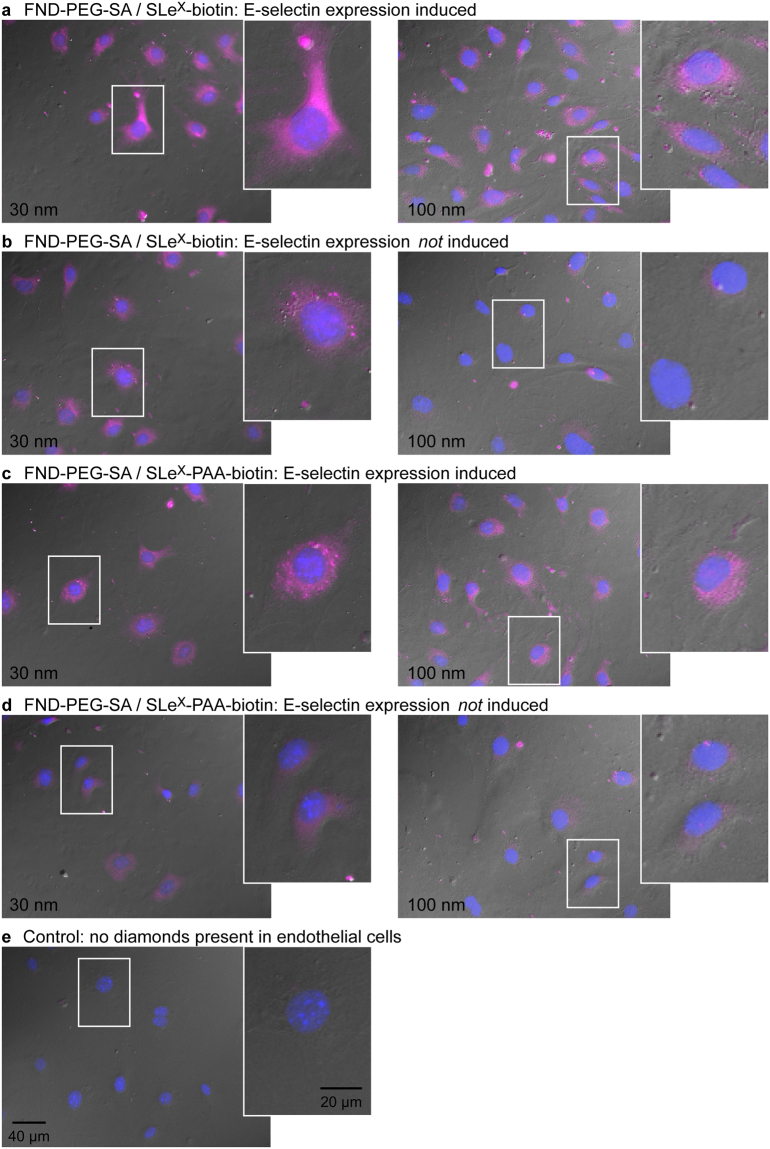
Binding of SLe^X^-conjugated 30 nm and 100 nm FND-PEG-SA samples to fixed mouse brain endothelial cells. Left: 30 nm FNDs, Right: 100 nm FNDs (**a**). FND-PEG-SA and the monovalent SLe^X^-biotin in cells expressing E-selectin (**b**) FND-PEG-SA and monovalent SLe^X^-biotin in cells not expressing E-selectin. (**c**) FND-PEG-SA and multivalent SLe^X^-PAA-biotin in cells expressing E-selectin (**d**) FND-PEG-SA and multivalent SLe^X^-PAA-biotin in cells not expressing E-selectin. (**e**) Endothelial cells without any nanodiamonds. All images were acquired using the same exposure time of 14 s on the FND channel. An overlay of DAPI (blue) and FND channels (pink) on the bright field image is shown. In each image, the section on each image indicated by a white box is magnified 2.5x.

Mouse brain endothelial cells (bEnd.3) were induced to express E-selectin using the inflammatory cytokine TNFα over a 4 hr incubation period. The 30 nm FNDs functionalized with both the mono (SLe^X^-biotin) - and multivalent (SLe^X^-PAA-biotin) SLe^X^ ligand were observed to bind within non-permeablized cells expressing E-selectin (*left column*, Fig. 2a,b – monovalent and Fig. 2c,d – multivalent). Cells with TNFα induced expression of E-selectin are shown in panels 2 A and 2 C. In Fig. 2, the FND-NV channel (555 nm, *colored pink*) is overlaid onto the DAPI channel (358 nm, *colored blue*) and the DIC image (shown in *grey*). DAPI binds strongly to DNA, thus staining the nucleus of cells.

The background autofluorescence level is negligible on the FND channel for cells that do not contain any nanodiamonds (or treatment with TNFα) (Fig. 2e). That is, the emission detected in the FND channel for Fig. 2a–d is due to FND fluorescence, and is free from any detectable background autofluorescence signal.

The cells expressing E-selectin (Fig. 2a,c, *left*) are visibly brighter in the FND channel when exposed to both SLe^X^-biotin and SLe^X^-PAA-biotin than in cells not stimulated by TNFα (Fig. 2b,d, *left*). The SLe^X^-biotin ligand (Fig. 2a, l*eft*) shows brighter staining than the other samples, suggesting that the monovalent ligand attached to the 30 nm FNDs is more accessible to the induced E-selectin molecules, compared to the multivalent ligand. When the uninduced (no TNFα) control cells are examined (Fig. 2b,d), there is still a significant level of FND binding, although at a decreased level, particularly for the monovalent ligand (Fig. 2b). This binding to the uninduced ‘control’ cells suggests the presence of basal levels of expressed E-selectin. This basal level expression of E-selectin has also been observed by others in proliferating cells^26^. The labeling of uninduced cells could also be due to the binding of the SLe^X^-biotin ligand to other competing selectin family CAM molecules, such as P-selectin, which is stored in granules within the endothelial brain cells^27^.

To compare the binding efficiency of the mono- and multivalent ligands, the signal intensity of emission on the FND channel (555 nm) was quantified for 10 individual cells in each sample. The average cell intensity for the binding of 30 nm FND-PEG-SA with SLe^X^-biotin and SLe^X^-PAA-biotin complexes inside cells in the presence and absence of TNFα is summarized in Table 2, and is also shown in Fig. 5a. Figure 5a shows that the brightness of the labeled cells is highly variable, as indicated by the large error bars on the data. This suggests a variable level of E-selectin expression, but nevertheless it is clear that TNFα stimulation resulted in a significant increased binding of both the monovalent and multivalent selectin ligand. It is apparent that when using either SLe^X^ ligand as the probe, the cells induced to express E-selectin were significantly brighter than the control uninduced cells (SLe^X^-biotin t-test: t(11) = 4.251, p-value = 0.00113, SLe^X^-PAA-biotin t-test: t(15) = 3.291, p-value = 0.00496). The brightest cells, which represent the highest level of FND binding, were observed in the 30 nm FND-PEG-SA/SLe^X^-biotin in the TNFα induced sample. While these cells were ~2 × brighter than the equivalent SLe^X^-PAA-biotin labeled cells, the ratio of cell brightness in the TNFα/No TNFα cells is essentially the same. This suggests that while there was increased binding using the SLe^X^-biotin ligand, this may have been due to an increase in the non-specific binding of the probe to another target, such as a CAM.

**Table 2:** The brightness of cells containing 30 nm FND-PEG-SA, 100 nm FND-PEG-SA, and SA-BHHTEGST-Eu complexed to E-selectin ligands SLe^X^-biotin or SLe^X^-PAA-biotin, in Arbitrary Units (AU) is listed. The brightness of cells containing 30 nm and 100 nm FND-PEG-E-selectin antibody (Ab) are also shown.

	30 nm FND-PEG			100 nm FND-PEG			SA-BHHTEGST-Eu	
	SA/SLe^X^-Biotin	SA/SLe^X^-PAA-Biotin	Ab	SA/SLe^X^-Biotin	SA/SLe^X^-PAA-Biotin	Ab	SLe^X^-Biotin	SLe^X^-PAA-Biotin
TNFα	27.6 ± 11.5	15.3 ± 7.6	16.0 ± 8.8	11.0 ± 7.0	13.7 ± 4.0	15.9 ± 8.1	10.4 ± 3.8	20.6 ± 9.5
No TNFα	10.9 ± 4.7	6.0 ± 4.7	0.4 ± 0.5	2.6 ± 3.9	5.2 ± 4.9	8.1 ± 2.3	7.5 ± 2.9	3.8 ± 1.5
TNFα/No TNFα	2.5	2.6	40	4.2	2.6	2.0	1.4	5.4

The average cell brightness intensity is presented (±standard deviation). The average background noise intensity was 0.04 for 30 nm FND-PEG-SA, 0.10 for 100 nm FND-PEG-SA, 1.28 for SA-BHHTEGST-Eu, and 0.01 for FND-PEG-Ab.

As a control for testing the binding affinity of the SLe^X^-biotin functionalized FNDs, we also tested the binding efficiency of the precursor FND. That is, the unmodified, carboxylated (FND-COOH) FNDs. The non-functionalized 30 nm carboxylated FNDs were observed to bind strongly to the endothelial cells (Supplementary Figure S5A). The average brightness of cells containing unmodified 30 nm FNDs was 32.7 ± 11.2 AU, comparable to the level of binding seen in the 30 nm FND-PEG-SA/SLe^X^-biotin sample (27.6 ± 11.5 AU). The non-specific binding of carboxylated FNDs to proteins and peptides has been reported by others^28^. It likely occurs due to the electrostatic interactions between the FND surface carboxyl groups and amine groups on proteins. The binding observed, while strong, does not provide information on the location of E-selectin within the cell. Information on the spatial distribution of E-selectin can be obtained by examining the binding of the targeted FNDs, that is, the FNDs functionalized with the mono- and multivalent ligands of E-selectin. E-selectin thus appears to be located throughout the cell cytoplasm, with increased concentration near the cell nuclei.

### Imaging of SLeX binding to E-selectin using 100 nm Fluorescent Nanodiamonds

The influence of the size of the FND on the binding efficiency was examined by comparing the binding of the 30 nm functionalized FNDs with 100 nm FND-PEG-SA probes coated with either the monovalent ligand SLe^X^-biotin, or the multivalent SLe^X^-PAA-biotin ligand (Fig. 2, *right panels*). FNDs functionalized with the monovalent ligand were found to bind to both the outer cell membrane and inside of cells expressing the target protein E-selectin (Fig. 2a, *right*). A similar level of binding was seen in TNFα cells using the 100 nm FNDs with the multivalent SLe^X^-PAA-biotin ligand (Fig. 2c, *right*). A small amount of non-specific binding of 100 nm conjugated FNDs can be seen within the control cells not induced to express E-selectin (Fig. 2b and d, *right*). It is important to note that the labeling of the cells with the 30 nm FNDs was predominantly observed to occur on the inside of the cell. In contrast, the cells labeled with the 100 nm FNDs bound to the cell interior and the cell surface.

To quantify the binding of the 100 nm FND probes to the cells, the average cell brightness was counted on individual cells as described previously for the 30 nm labeling experiments (Fig. 5b and Table 2). The standard deviation of cell brightness was again large across the samples tested, and again there is an increase in binding of both SLe^X^ biotin ligands to E-selectin upon their TNFα stimulation. For both ligands, the cells induced to express E-selectin were found to be significantly brighter than the uninduced cells (SLe^X^-biotin t-test: t(14) = 3.315, p-value = 0.00511, SLe^X^-PAA-biotin t-test: t(17) = 4.249, p-value = 0.000541). For the 100 nm multivalent FND-PEG-SA/SLe^X^-PAA-biotin sample, the brightness of cells induced to express E-selectin was similar to the multivalent 30 nm FND-PEG-SA/SLe^X^-PAA-biotin sample, with average values of 15.3 ± 7.6 AU and 13.7 ± 4.0 AU, respectively. For the monovalent SLe^X^-biotin ligand, the 30 nm FNDs were 2.5 × brighter than the 100 nm FNDs in cells expressing E-selectin (27.6 ± 11.5 AU and 11.0 ± 7.0 AU, respectively). The binding level of the 30 nm FND-PEG-SA/SLe^X^-biotin was substantially greater than that of the 100 nm FND-PEG-SA/SLe^X^-biotin ligand (t-test: t(14) = 3.899, p-value = 0.00113). This is noteworthy considering that the 30 nm FNDs contain approximately 3 NV centers per particle, while the 100 nm FNDs contain approximately 500 NV centers per particle. That is, the 100 nm FNDs are ~170 × brighter than the 30 nm FNDs.

Similar to the 30 nm FNDs, 100 nm unmodified carboxylated FNDs (FND-COOH) were found to bind non-specifically to endothelial cells (Supplementary Figure S5B), where the average brightness of cells containing unmodified 100 nm FNDs was 17.9 ± 7.5 AU. Surprisingly, this was brighter than that seen with the SLe^X^-biotin ligand functionalized 100 nm FNDs, where a maximum cell brightness of 13.7 ± 4.0 AU was observed for FND-PEG-SA/SLe^X^-PAA-biotin binding to TNFα-induced cells. The binding of the 100 nm FND-COOH again does not indicate the location of E-selectin, since the unmodified FNDs are not designed to target the CAM molecule. Since the level of non-specific binding is highest in the unmodified FNDs, the functionalization of FNDs with glycan ligands of E-selectin effectively reduces the non-specific binding of 100 nm FNDs.

### Imaging of SLe^X^ binding to E-selectin using time gated luminescent microscopy with an Europium chelate

**Figure 3:**
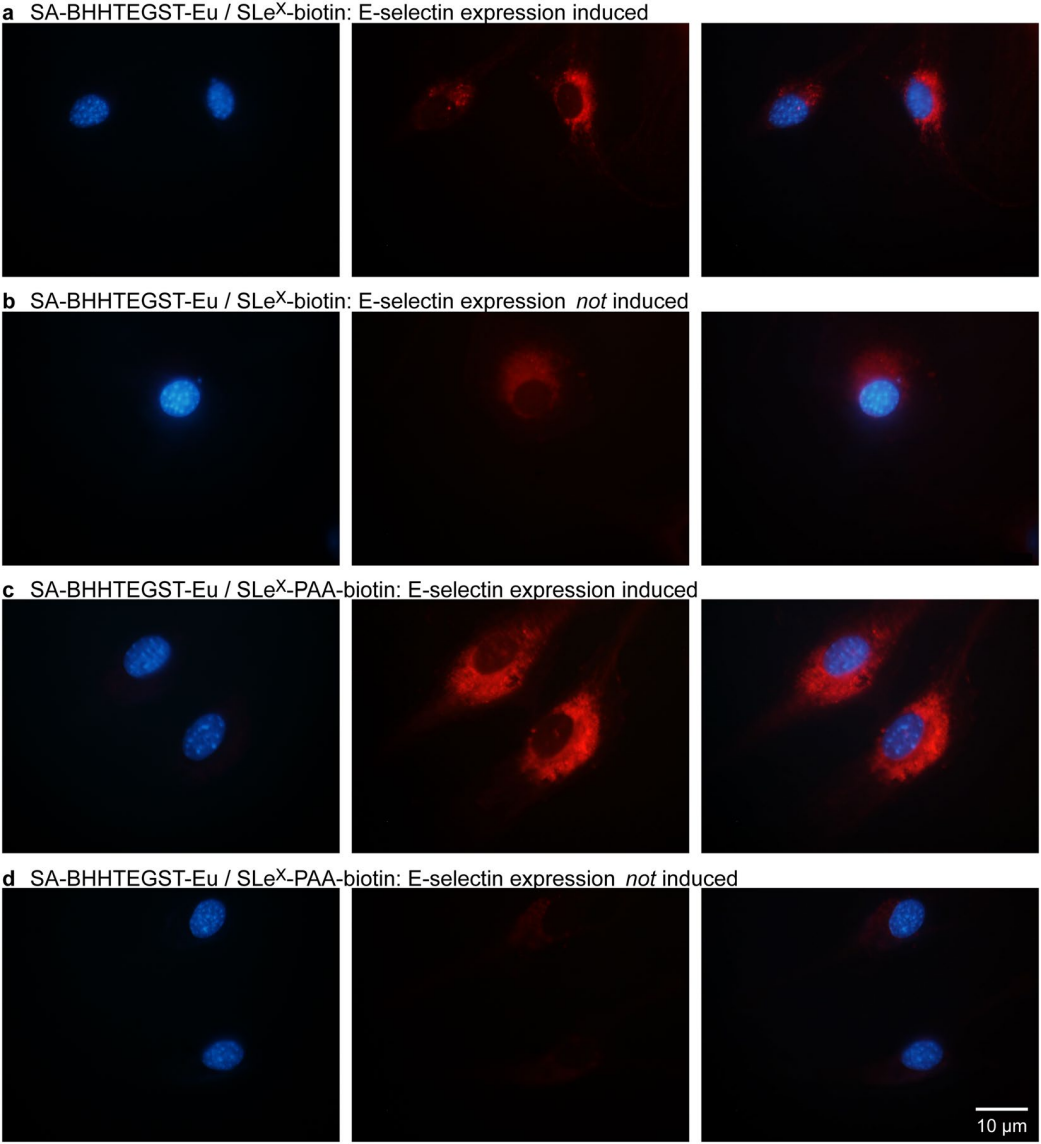
Binding of SLe^X^-conjugated SA-BHHTEGST-Eu samples to fixed mouse brain endothelial cells. Left: DAPI, middle: Eu Red luminescence TGL image, right: overlay (**a**) SA-BHHTEGST and the monovalent SLe^X^-biotin in cells expressing E-selectin (**b**) SA-BHHTEGST and the monovalent SLe^X^-biotin in cells not expressing E-selectin. (**c**) SA-BHHTEGST and the multivalent SLe^X^-PAA-biotin in cells expressing E-selectin (**d**) SA-BHHTEGST and the multivalent SLe^X^-PAA-biotin in cells not expressing E-selectin. TGL Images were captured using identical exposure times (5.0 s).

A SA-BHHTEGST-Eu probe (Fig. 1aii) was also examined for visualization of E-selectin with the SLe^X^ ligand. Time-gated luminescent (TGL) microscopy is alternate approach to minimizing background autofluorescence. The SA-BHHTEGST-Eu bound to the monovalent (SLe^X^-biotin) and multivalent SLe^X^ (SLe^X^-PAA-biotin) ligands of selectin were used to acquire time-gated luminescence images of binding of the SLe^X^ to the TNFα induced cells (Fig. 3).

Like the 30 nm FNDs probes, the small SA-BHHTEGST-Eu probe could readily access the interior of the fixed cell, thus showing the cytoplasmic location of endocytosed E-selectin. There was no significant difference in the brightness of induced (Fig. 3a and Table 2) and uninduced cells (Fig. 3b and Table 2) targeted with the monovalent SLe^X^ ligand (t-test: t(16) = 1.921, p-value = 0.0716). However, the induced cells targeted with the multivalent ligand (Fig. 5c) were significantly brighter than the uninduced cells (Fig. 3d), (t-test: t(9) = 5.489, p-value = 0.000386). Comparison of the monovalent ligand (Fig. 3a,b) with the multivalent ligand (Fig. 3c,d) shows that, in contrast to the conjugated 30 nm FNDs, and similarly to the 100 nm FNDs, the multivalent ligand (Fig. 3c,d) was able to detect E-selectin with a substantially higher sensitivity than the monovalent ligand (Fig. 3a,b) after stimulation with TNFα.

Comparison of the cells to which SA-BHHTEGST-Eu/SLe^X^-PAA-biotin was added shows a clear difference between the TNFα-induced and uninduced cells (Fig. 5c,d), where the induced cells were 5.4 × brighter than the uninduced cells, on average (Table 2). The Eu chelate labeling using the SLe^X^ ligands displayed in the largest difference in signal between induced and uninduced cells. The multivalent presentation of the biotinylated SLe^X^ ligand on the PAA backbone potentially amplifies the labeling of a single E-selectin by binding to more than one SA-BHHTEGST-Eu molecule (Fig. 1b). As observed when the targeted 30 nm FNDs were tested (Fig. 2), there appears to be a concentration of E-selectin molecules close to the nucleus in the cytoplasm of cells. This is most apparent in the induced cells to which SLe^X^-biotin- SA-BHHTEGST-Eu was added (Fig. 3c).

The standard deviation of the cell intensity for these experiments was again large. This variability was observed for all the labels tested (30 nm FNDs, 100 nm and SA-BHHTEGST-Eu), and is likely a result of the variable expression of E-selectin (or possibly a variation in the expression levels of other recognized targets of SLe^X^, such as the CAM, P-selectin^27^).

### Antibody-coated FNDs targeting E-selectin

To confirm the binding of the SLe^X^ ligands as indicators of E-selectin induction by TNFα, the 30 and 100 nm FNDs were conjugated to an antibody specific for E-selectin. EDC and NHS reagents were used to attach the antibody to PEGylated 30 and 100 nm sized FNDs as outlined in the experimental section (FND-PEG-Ab). Unlike the SLe^X^ ligand which is able to bind to both E- and P-selectin, the antibody used for these experiments binds only to E-selectin. This FND antibody construct was applied to cells with and without E-selectin induction by TNFα, such that the conditions of the experiments performed for the SLe^X^ ligand conjugated FNDs were replicated.

**Figure 4:**
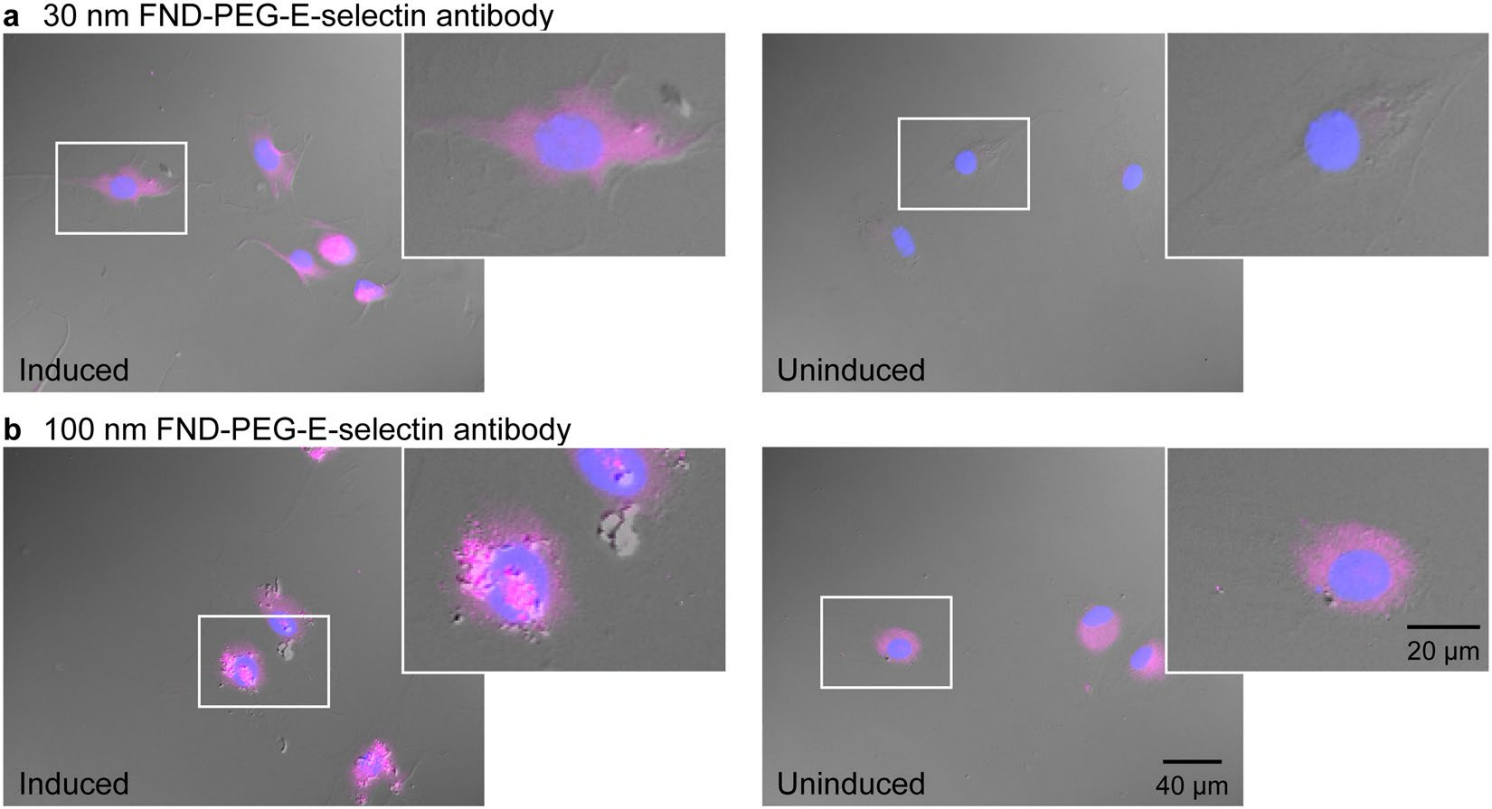
Binding of 30 nm and 100 nm FNDs coated with PEG and the E-selectin antibody (FND-PEG-Ab) to fixed mouse brain endothelial cells. (**a**) 30 nm FND-PEG-Ab in cells induced to express E-selectin (left) and in uninduced cells (right). (**b**) 100 nm FND-PEG-Ab in cells expressing E-selectin (left) and in uninduced cells (right). An overlay of DAPI (blue) and the FND channel (pink) is shown. All images were acquired using the same exposure time of 14 s on the FND channel. In each image, the section on each image indicated by a white box is magnified 2.5x.

Cells were induced to express E-selectin using TNFα for 4 hrs, a time period found to maximize E-selectin expression^27^. The basal levels of E-selectin expression were also determined by testing the conjugated Ab without cytokine stimulation. The 30 nm FND-PEG-Ab did not bind detectably to the uninduced cells, whereas high levels of binding of the Ab probe were seen in the TNFα induced cells (Fig. 4a). The location of the 30 nm FND-PEG-Ab was predominantly within the cell, similar to that seen with the 30 nm glycosylated ligand conjugated FNDs.

**Figure 5:**
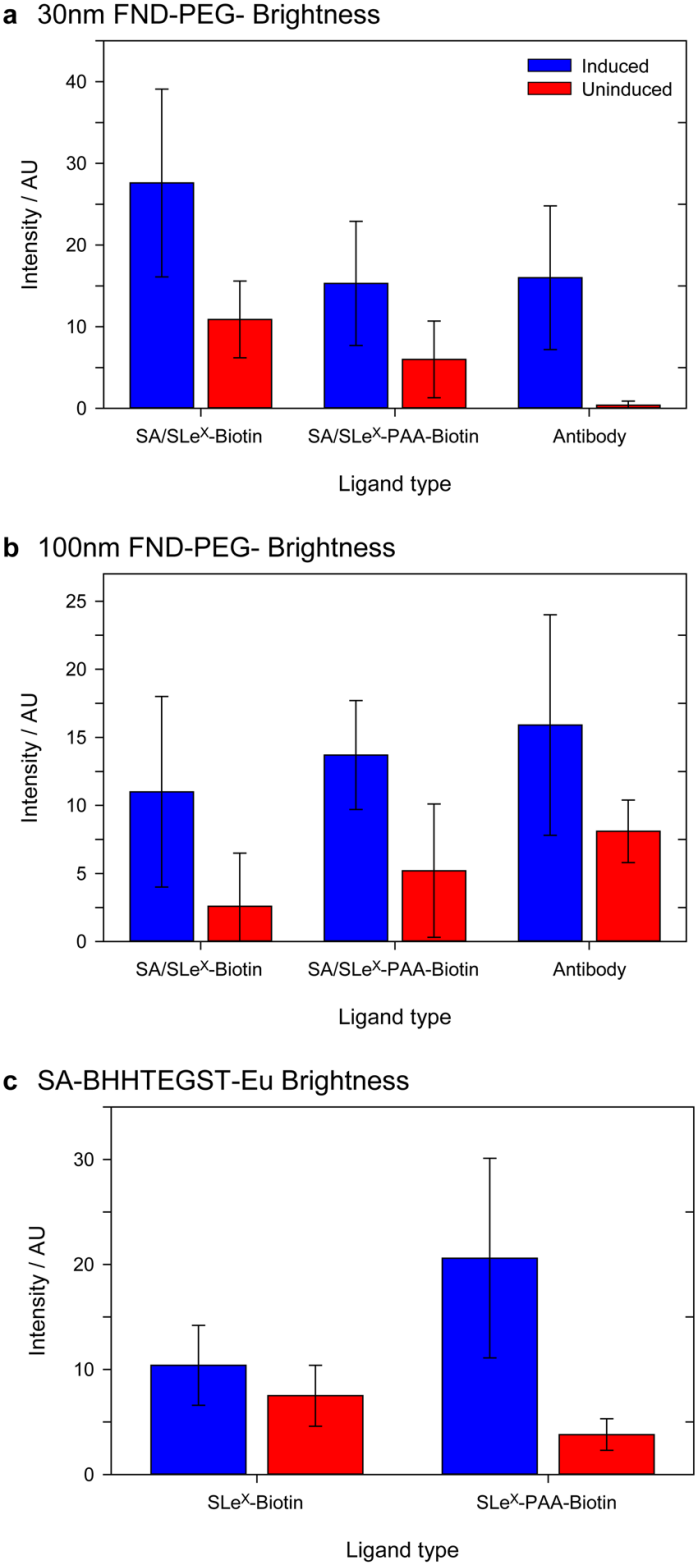
The signal intensity of cells on the detected channel is illustrated. The average intensity of 10 cells on each slide is presented, where error bars indicate the standard deviation of the 10 measured cells. The average brightness of cells in which E-selectin expression was induced with TNFα are colored blue, and those which were not induced to express E-selectin are colored red. (**a**) 30 nm FND-PEG-SA used with monovalent SLe^X^-biotin and multivalent SLe^X^-PAA-biotin, compared to the antibody-functionalised FNDs of the same size. (**b**) 100 nm FND-PEG-SA used with monovalent SLe^X^-biotin and multivalent SLe^X^-PAA-biotin, compared to the antibody functionalised nanodiamonds. (**c**) SA-BHHTEGST-Eu used with monovalent SLe^X^-biotin and multivalent SLe^X^-PAA-biotin.

The brightness of cells containing the antibody-coated FND probe was also quantified (Table 2, Fig. 5). For the TNFα-induced cells, the average brightness observed for the 30 nm FND-PEG-Ab (16.0 ± 8.8 AU) was similar to that seen in the 100 nm FND-PEG-Ab (15.9 ± 8.1 AU). Both the 30 nm and 100 nm FND-PEG-Ab samples were significantly brighter in the induced cells, compared to the uninduced cells (30 nm t-test: t(9) = 5.597, p-value = 0.000336, 100 nm t-test: t(10) = 2.929, p-value = 0.0151).

In contrast to the 30 nm FND-PEG-Ab, and like the 100 nm SLe^X^ glycosylated FNDs, the 100 nm FND-PEG-Ab probes were found to bind both inside and on the surface of the cells expressing E-selectin (Fig. 4b). There was also a notable clumping of the 100 nm FNDs on the cell membrane. While the clumping of FNDs to the cell membrane was not observed in the uninduced cells (Fig. 4b, right), there is clearly a substantial number of FNDs inside the cells before TNFα induction, with an average cell brightness of 8.1 ± 2.3 AU. The detection of E-selectin in uninduced cells using the larger 100 nm FND-PEG-Ab, but not the 30 nm FND-PEG-Ab, is a curious result that may be due to the substantially increased inherent NV brightness of the 100 nm FNDs, compared to the 30 nm FNDs. Since 100 nm FNDs are ~170 × brighter than 30 nm FNDs, it is possible to detect a smaller number of these larger FNDs.

## Discussion

In this study, we have demonstrated two different probe based approaches for reducing cellular background autofluorescence when imaging highly autofluorescent brain endothelial cells. E-selectin binding in these cells was easily detected with the natural ligand SLe^X^ attached via a biotin/SA conjugation to (1) fluorescent nanodiamonds of various diameters and (2) a europium-based BHHTEGST chelating system. Both these approaches were able to detect inflammation in the cells after TNFα stimulation. Antibody conjugated FNDs were also targeted to E-selectin. Of all the probes examined, the highest sensitivity of detection was achieved using 30 nm FNDs conjugated to an anti-E-selectin antibody. While 100 nm FNDs are naturally brighter emitters than 30 nm FNDs, they are also inherently more limited by diffusion for access to their targets, and may be restricted in their ability to freely pass through the cell membrane for intracellular binding. Smaller probes, such as 30 nm FNDs or the SA-BHHTEGST construct, are more able to freely diffuse in solution and bind to targets.

E-selectin is a key protein upregulated in inflamed brain endothelial tissues, including vascular disorders, after gamma radiation knife surgical treatment^29^. E-selectin expression can also be induced using inflammatory cytokines such as TNFα, as is P-selectin^27,30^. While E-selectin is functional as a membrane bound glycoprotein, it is rapidly internalized into the cytoplasm^31,32^ via clathrin mediated endocytosis^33^. This is consistent with our observations here where we found mostly cytoplasmic staining of E-selectin. The presence of E-selectin on the cell membrane was also detected using 100 nm antibody-coated FNDs, but the signal was minimal. It is likely that the larger sized 100 nm FND-PEG-Ab complex had limited access into the fixed cells to some degree, thus explaining the clustering of the FNDs on the cell surface.

Cell staining using the 30 nm FNDs coated with the E-selectin antibody had the highest specificity and sensitivity (Fig. 4), where a 40-fold increase in brightness was observed for cells treated with TNFα, in comparison to uninduced cells. Of the probes utilizing the SLe^X^ glycan ligand, the 30 nm monovalent FND-PEG-SA/SLe^X^-biotin sample resulted in the strongest levels of binding in the induced cells in comparison to the SA-BHHTEGST-Eu/SLe^X^-biotin construct. On the other hand, the SA-BHHTEGST-Eu/SLe^X^-PAA-biotin construct was more advantageous in differentiating E-selectin levels in TNFα induced cells compared to the control uninduced cells, since the induced cells were on average 5-fold brighter than the uninduced cells (Table 2). However, it should also be noted that unlike nanodiamonds, Eu-chelating tags readily photobleach, which is problematic for *in vivo* applications such as targeting of biomolecules for live cell tracking. FNDs do not photobleach and so are superior probes for the infinite tracking of biological species. The lack of toxicity of FNDs is also highly advantageous for these applications^5^.

The 100 nm FND-PEG-SA showed better cellular binding when used with the multivalent SLe^X^-PAA-biotin ligand. The reason as to why the monovalent SLe^X^ ligand was better with 30 nm FNDs and the multivalent SLe^X^-PAA-biotin ligand better with 100 nm FND is not immediately clear. It is possible that the relative size of the multivalent ligand, compared to the monovalent ligand, reduced the mobility of the 30 nm FND-PEG-SA, thus limiting the efficiency of binding to E-selectin for this construct. For the 100 nm FNDs, the size of the multivalent adduct could have had less of an impact on the dynamic radius of the already large 100 nm FNDs. For the SA-BHHTEGST-Eu probe construct, a greater sensitivity of E-selectin detection was also observed with the multivalent SLe^X^ ligand and not the monovalent ligand. For these smaller soluble Eu chelated tags, the multiple biotins attached to the multivalent SLe^X^ ligand enabled amplification of the fluorescent signal, since many SA-BHHTEGST-Eu/SLe^X^ units could be linked together at the location of a single E-selectin molecule. Conversely, when the monovalent ligand is utilized, only one SA-BHHTEGST-Eu/SLe^X^-biotin construct is capable of binding to each E-selectin molecule.

Some binding of the FND and lanthanide constructs to brain endothelial cells that were not exposed to TNFα was observed, which may be due to basal levels of E-selectin expression during their normal cell cycle^26^. Other members of the selectin family (L-selectin and P-selectin) also bind to Sialyl Lewis motifs. Both P- and E-selectin are expressed on endothelial cells, whereas L-selectin is expressed on leukocytes^27^. While SLe^X^ is the natural ligand of E-selectin, this glycan motif is also capable of binding to P-selectin in endothelial cells, resulting in additional probe binding, although the 4 hour TNFα induction condition has been shown to favor E-selectin expression over P-selectin^27^. P-selectin is stored in granules within endothelial cells so that upon induction, expression on the cell surface is rapid. The maximal expression of E-selectin, on the other hand, peaks 3–4 hours after induction with TNFα^27^. Therefore, it is more likely in our experiments that E-selectin was the observed binding target of our glycan-functionalized FNDs and Eu-chelates four hours following TNFα application. This was confirmed by using the FNDs conjugated to the E-selectin specific antibody.

Endogenous biotin is expressed in many types of cells^34^, and this could also account for some ‘non-specific’ binding of the biotinylated probes observed in the control cells that were not induced to increase E-selectin expression. Since each SA molecule is able to bind to four biotins, it is possible that ‘free’ biotin binding sites were present on SA molecules after attachment to SLe^X^ moieties. While commercial kits are available to decrease the non-specific binding due to endogenous biotin in cells, we found that the problem could be best minimized by using a large excess of SLe^X^ biotinylated ligand species. Regardless, due to steric constrictions, particularly pertinent to the bulky multivalent SLe^X^-PAA-biotin species, it is possible that endogenous biotin within the fixed cell could have bound to the SA-complexes to yield some non-specific binding interaction although this was minimal.

In summary, we have shown that both fluorescent nanodiamonds and europium chelating tag based probes efficiently target the inducible cellular protein E-selectin via an antibody or by using a biotin/streptavidin-based ligand scaffold. This scaffold allows for the rapid selection of a target with any known biotinylated ligand. Both types of probe developed in this study provided background free imaging. While the utilization of small molecule ligands, such as SLe^X^ in this case, can be strategic with regards to the size of the targeting molecule, ultimately the highest degree of specificity observed in this study was obtained using specifically targeted antibodies, as demonstrated with our 30 nm FND-PEG-Ab construct. Although 100 nm FNDs were brighter than 30 nm FNDs, the best result for the selective illumination of E-selectin in endothelial brain cells was obtained using 30 nm FNDs, highlighting the importance of particle size for nanodiamond-based imaging - bigger and brighter nanodiamonds are not always better.

## Methods

### Nanodiamond material

The fluorescent nanodiamonds (FND) used in this experiment were synthetic type Ib diamond powders. The FND powder was treated and processed as previously described^35^. 30 nm FNDs (Adamas Nano) were estimated to contain 1–3 NV centers per particle; and 100 nm FNDs (Academia Sinica) were estimated to contain ~500 NV centers per particle^12,36^.

### Nanodiamond conjugation

#### PEGylation of fluorescent nanodiamonds: FND-PEG

30 nm or 100 nm carboxylated fluorescent nanodiamonds (0.8 mg) were suspended in acidic (pH 4) double distilled water (DDW). The carboxyl groups were activated by incubating the FNDs with *N*-(3-dimethylaminopropyl)-*N′*-ethylcarbodiimide hydrochloride (EDC, 3.2 mg) and *N*-hydroxysuccinimide (NHS, 4.8 mg) for 20 mins. All steps were performed at room temperature unless otherwise indicated. The EDC was inactivated through the addition of dithiothreitol (DTT) to a final concentration of 50 mM.

After centrifugation to pellet NDs (16,000 × g, 25 mins), the activated FNDs (FND-NHS) were resuspended in basic DDW (pH 8). The FND-NHS’s were then incubated with poly(ethylene glycol) 2-aminoethyl ether acetic acid (CA(PEG)_22_, 4 mg) for 3 hrs under agitation (3,000 rpm). Incubation of the FND-PEG reaction was continued at 4 °C for a further 16 hrs. Ethanolamine (50 mM) was then added to quench the reaction through its reaction with any remaining NHS esters present on the FNDs. Following 2 hrs of incubation, excess CA(PEG)_22_ and ethanolamine were removed by centrifugation (16,000 × g, 25 mins), and the final FND-PEG pellet resuspended in neutral DDW (pH 7).

#### Conjugation of Streptavidin to fluorescent nanodiamonds: FND-PEG-SA

The terminal carboxyl groups on the PEG spacer arm were activated with EDC and NHS (as described above) before its reaction with 100 μg of recombinant streptavidin (SA) (*Streptomyces Avidinii*) in basic DDW (pH 8). Again, the reaction was incubated at for 3 hrs (with agitation at 3000 rpm), then incubation continued for a further 16 hrs at 4 °C before ethanolamine (50 mM) was added to quench the reaction.

The final FND-PEG-SA product (Fig. 1ai, right) was washed and resuspended in neutral DDW at a final concentration of 2 mg/mL. FND-PEG-SA was diluted into PBS buffer (pH 7.4) containing 1 mg/mL BSA to form a final FND concentration of ~0.5 mg/mL. The concentration of FND was determined by the linear fluorescent emission at 678 nm after excitation at 530 nm (Fluorolog, Em_int_ = 2242.9 × [100 nm FND μg/mL] − 86850). Dynamic Light Scattering (DLS) size distributions and zeta potential measurements were obtained before and after each reaction step using a Zetasizer Nano ZS with a 633-nm laser source supplied by Malvern Instruments (Supplementary Figure S1). The size distributions of the particles were measured over an average of 100 scans each 30 sec. long, and the zeta potentials were measured over 300 scans. Both size distribution and zeta potential were determined using a back-scattering configuration (173°). Size distribution data were reported as number-weighted statistical distributions. Confirmation of the successful attachment of the streptavidin coating to the FND scaffold was further demonstrated by reaction of the FND-PEG-SA complex with biotinylated FITC (1 hr, 3000 rpm). The resulting complex was then examined using confocal microscopy (Supplementary Figure S2).

#### Conjugation of E-selectin antibody to fluorescent nanodiamonds: FND-PEG-Ab

The E-selectin antibody (recombinant human E-selectin Fc chimera, R&D Systems 724-ES) was also conjugated to both the 30 nm and 100 nm FND-PEG scaffolds using the same procedure described above. DLS size distributions and zeta potential measurements were performed before and after the conjugation were used to assess the successful conjugation of E-selectin antibody to the FND-PEG (FND-PEG-Ab).

### Conjugation of Streptavidin to BHHTEGST

We have recently developed a novel europium chelate, BHHTEGST-Eu (4,4′-bis(1′′,1′′,1′′,2′′,2′′,3′′,3′′-heptafluoro-4′′,6′′-hexanedion-6′′-yl)sulfonylamino-tetraethyleneglycol-succinimidyl carbonate-o-terphenyl)) that has enhanced aqueous solubility^13^. The detailed procedure of the conjugation of europium chelate to streptavidin (SA-BHHTEGST-Eu), purification and the optimal molar ratio of europium chelate per streptavidin was previously reported^14^. Briefly, 100 μg of streptavidin in 100 mM NaHCO_3_, pH 8.5 was mixed with a 20-fold molar excess of the BHHTEGST chelate. After incubation for 1 h at 37 °C, the reaction mixture was passed through a Sephadex G-25 column (PD MiniTrap) using 0.1× PBS (0.01% v/v Tween 20) to elute the purified conjugated protein (SA-BHHTEGST), free from unbound chelate. The fractions containing purified SA-BHHTEGST were collected according to absorbance detection using an Eppendorf BioPhotometer (280 and 320 nm) (Fig. 1aii).

UV-visible absorption analysis of BHHTEGST (NanoDrop UV spectrometer) indicated a maximum UV absorption at 335 nm and also partial absorption at 280 nm which overlaps with that of the SA. To evaluate the partial absorption of BHHTEGST moiety in the conjugated SA, the molar extinction coefficient of the chelating tag at 335 nm and 280 nm were separately obtained from UV-visible analysis of purified BHHTEGST [ε_335_ = 3.14 × 10^4^ M^−1^ cm^−1^, ε_280_ = 1.75 × 10^4^ M^−1^ cm^−1^]^13^. The concentration of the BHHTEGST was then obtained by reading the absorbance of conjugates at 335 nm (assuming that the extinction coefficient of BHHTEGST does not change on the labeled antibody). SA concentration was obtained by subtracting the absorbance of BHHTEGST from the absorbance of labelled protein at 280 nm. The number of BHHTEGST molecules per streptavidin then was obtained by dividing the molar ratio of ligand to streptavidin^13,14^.

### Addition of biotinylated Sialyl Lewix X: SLeX-biotin and SLeX-PAA-biotin

The two biotinylated constructs of Sialyl Lewis X (SLe^X^) were purchased from Lectinity: (i) monovalent SLe^X^-biotin and (ii) multivalent SLe^X^-PAA-biotin. The multivalent ligand consisted of 20% (mol/mol) SLe^X^ units and 5% biotin on a poly-N-(2-hydroxyethyl)acrylamide backbone^37^. 100 μL of the FND-PEG-SA (0.5 mg/mL) scaffold was mixed with SLe^X^-biotin (Fig. 1bi) or SLe^X^-PAA-biotin (Fig. 1bii) (10 uL, 0.1 mg/mL) in PBS buffer containing 1 mg/mL BSA. After 1 hr of incubation (3000 rpm shaking), the excess SLe^X^ glycan was removed by centrifugation.

50 μL of the SA-BHHTEGST scaffold (0.2 mg/mL) was also separately mixed with SLe^X^-biotin or SLe^X^-PAA-biotin (10 uL, 0.1 mg/mL) in PBS buffer containing 1 mg/mL BSA, and then incubated for 10 mins.

### Quantification of SLe^X^ on FND-PEG-SA constructs

Fluorescein labeled Maackia Amurensis Lectin I (MAL 1-FITC), a lectin that binds to the SLe^X^ motif, was incubated with 100 nm FND-PEG-SA/SLe^X^- biotin and 100 nm FND-PEG-SA/SLe^X^-PAA-biotin for 2 hrs (under 3000 rpm of shaking) according to manufacturer’s instructions (Vector Laboratories). The unbound MAL 1-FITC lectin was removed by centrifugation. The concentration of FND and MAL 1-FITC in each sample was determined using standard solutions. Standard curves to determine FND concentration were constructed from fluorescence emission values at 678 nm, after an excitation at 530 nm (Supplementary Figure S3). Standard curves to determine the concentration of MAL 1-FITC were based on the FITC fluorescence emission at 515 nm, after its excitation at 490 nm (Supplementary Figure S4).

### Cell culture

Mouse brain endothelial (bEnd.3[BEND3]; ATCC® CRL2299™) cells were maintained in DMEM medium supplemented with 10% (v/v) fetal calf serum, 50 IU/mL penicillin, and 50 μg/mL streptomycin. Cells were grown until passage 32 as E-selectin expression is typically optimal at passage numbers greater than 30, as per the manufacturer’s protocol.

Cells were seeded at a density of 10^4^ cells/cm^2^ onto 12-well plates containing 12 mm round coverslips (ProSciTech). After 24 hours of incubation at 37 °C (5% CO_2_ v/v), expression of E-selectin was induced with the addition of 20 ng/mL Tumor Necrosis Factor α (TNFα). TNFα was added to 6 of the 12 wells. Incubation at 37 °C and 5% CO_2_ (v/v) was continued for another 4 hrs^38^. After this 4 hrs of expression, cells were fixed using 4% (v/v) paraformaldehyde for 15 mins. Cells were then washed with PBS and stored in PBS until used for cell staining (within 24 hrs).

### Cell-staining

After removing the storage buffer, 1 mL of PBS was placed in each well of a 12-well plate containing the fixed, non-permeablized bEnd.3 cells on coverslips. 5 μL of 0.5 mg/mL of either: (i) FND-PEG-SA/SLe^X^-biotin, (ii) FND-PEG-SA/SLe^X^-PAA-biotin, (iii) SA-BHHTEGST/SLe^X^-biotin, (iv) SA-BHHTEGST/SLe^X^-PAA-biotin, or (v) FND-PEG-Ab were then added to wells of the plate. After 2 hrs of incubation at 37 °C (500 rpm shaking), any unbound fluorescent probe material was removed from the wells. The wells were washed three times with 2 mL of PBS (5 mins per wash, 750 rpm shaking). For experiments involving FND-PEG-Ab, cells were first blocked by incubation with normal horse serum for 30 mins (100 μL/mL).

For all experiments, control samples were prepared in the same manner using cells that were not induced by TNFα to express E-selectin, but were incubated in parallel for the same time period. After washing wells with PBS, a final wash with DDW was performed. The 12 mm coverslips inside each well containing FNDs were mounted using 15 μL of ProLong® Gold with DAPI nuclear stain (Thermo Fisher). The coverslips inside the wells containing SA-BHHTEGST were mounted with 5 µL of europium chloride [EuCl_3_, 20 mM in fluorescence enhancing buffer (FEB)^39^] in addition to 15 μL of ProLong® Gold with DAPI nuclear stain. All coverslips were sealed immediately onto the slide with nail varnish and dried overnight at room temperature before their imaging.

### Imaging of the cells

Slides containing FNDs were photographed using wide-field microscopy on an Olympus BX63 fluorescent microscope. A custom installed filter (Olympus) suitable for the detection of florescent emission from nanodiamond NV centers was used: 555 nm excitation 25 nm bandwidth (ET555/25 × Chroma); 635 nm dichroic (T635lpxr Chroma); and 700 nm emission 75 nm bandwidth (ET700/75 m Chroma). Cells were photographed with a 40 × 1.6 numerical aperture dry objective with a DP80 dual monochrome/color camera. The exposure time was kept constant at 14 s on the NV channel for all samples.

For cells labeled with BHHTEGST-Eu, all bright-field, DAPI and time-gated luminescence (TGL) imaging was performed on an Olympus BX51 upright fluorescence microscope. Time-gated luminescence was performed using a Gated Auto-synchronous Luminescence Detector (GALD)^40^, developed previously by the research team’s faculty, which was inserted into the differential interference contrast (DIC) slot of the microscope. Time-gated luminescence images were captured without a fluorescence filter using a DP72 color camera set for ASA speed of 200 and exposure period of 5.0 s; all images were stored as TIFF files as captured.

### Quantification of cell brightness

The brightness of cells containing FNDs or BHHTEGST-Eu, cells was quantified based on the average pixel intensity on the relevant fluorescence channel, using the Zen Blue image analysis module (Zeiss). The average relative brightness, in arbitrary units (AU), and the standard deviation of brightness for a total of 10 cells observed on each sample slide was calculated. Regions not containing cells were also selected and analyzed, and the average intensity measurement of these areas was used to calculate the image’s noise intensity. For calculations of the signal-to-noise ratio, the average brightness value was divided by the average noise intensity of the sample.

Two-sample t-tests were performed to compare the mean brightness of samples, which were found to follow normal distributions. Test statistic values, degrees of freedom and two-sided p-values are reported.

### Data availability

The images and datasets generated and analyzed during the current study are available from the corresponding author on reasonable request.

## Electronic supplementary material

Supplementary Information
