# Supplementary material for: Reduced background autofluorescence for cell imaging using nanodiamonds and lanthanide chelates
Source: Sci Rep. 2018 Mar 14;8:4521. doi: 10.1038/s41598-018-22702-1 (PMC5851999; doi:10.1038/s41598-018-22702-1)
Supplement: Supplementary file 1 — Supplementary Information [file 41598_2018_22702_MOESM1_ESM.pdf]

Supplementary Information

100nm Sample	Intensity (678 nm)	FND conc (μg/mL)	Intensity (515 nm)	MAL1-FITC (μg/mL)	MAL1/ND ratio
SLe^{X}-biotin	74197	226	135454	0.121	0.00054
SLe^{X}-PAA-biotin	67510	188	110439	0.094	0.00050

Table S1. Quantification of MAL 1-FITC bound to 100 nm FND-PEG-SA samples with either the monovalent (SLe^{X}-biotin) or multivalent (SLe^{X}-PAA-biotin) E-selectin ligand attached to the surface. Samples excited at 532 nm and emission monitored at 678 nm were used to interpolate the FND concentration. Samples were then excited at 495 nm and emission recorded at 515 nm to interpolate the concentration of MAL1-FITC.

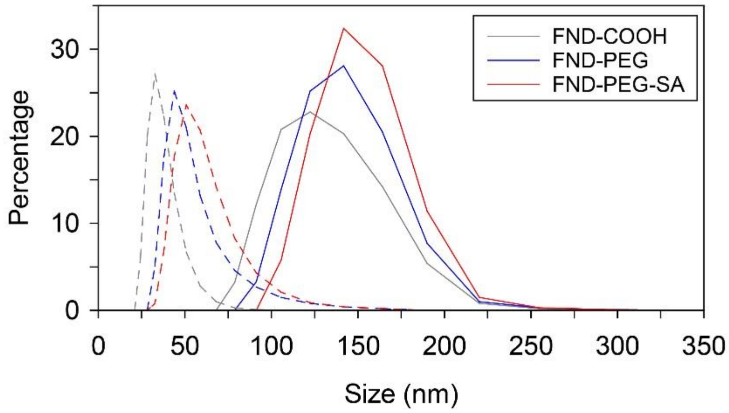

Figure S1: DLS size distributions (number-weighted) of 30 nm (dashed series) and 100 nm (solid lines) FNDs at each stage of the conjugation. The fraction of the number distribution is plotted against particle size.

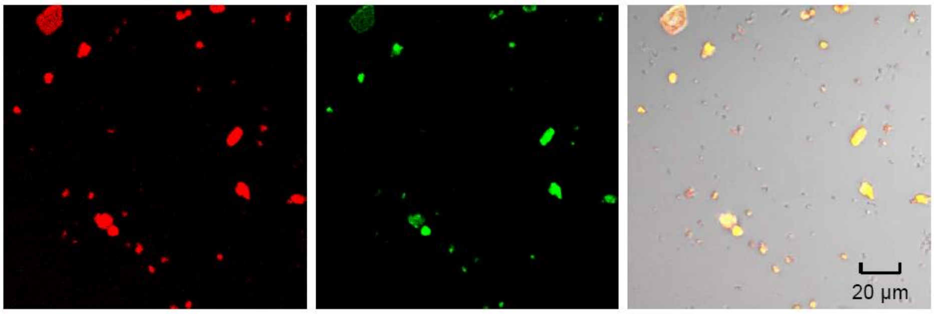

Figure S2. Colocalisation experiment to assess the surface coating of 100 nm FNDs with PEG and streptavidin. 100 nm FND-PEG-SA samples were incubated with biotinylated-FITC. *Left:* FND-NV channel (red), *middle:* FITC channel (green), *right:* overlay of FND-NV and FITC channels on the bright-field image. The yellow observed in the overlay indicates colocalisation of FND-NV fluorescence (red) with the biotin-FITC (green) signals.

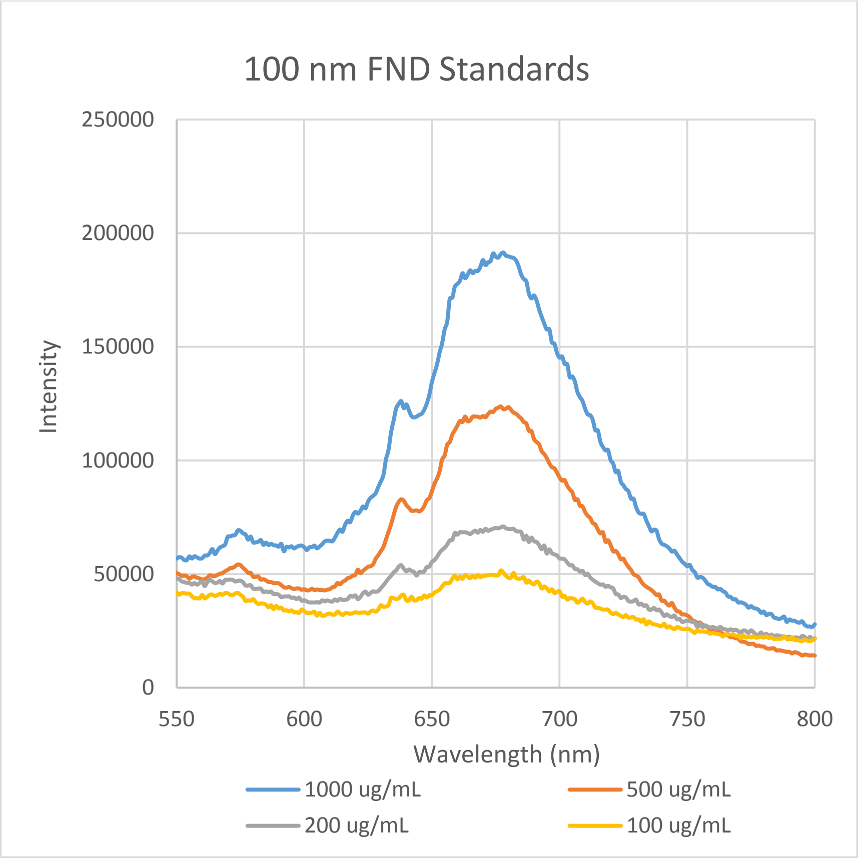

Figure S3. Emission spectra of 100 nm FND standard solutions used to determine the FND concentration of samples conjugated to Sialyl Lewis X (SLe^{X}). The concentration of each FND standard is displayed below the graph. A standard curve was constructed using the emission maxima of each standard (at 678 nm). Standards were excited at 532 nm.

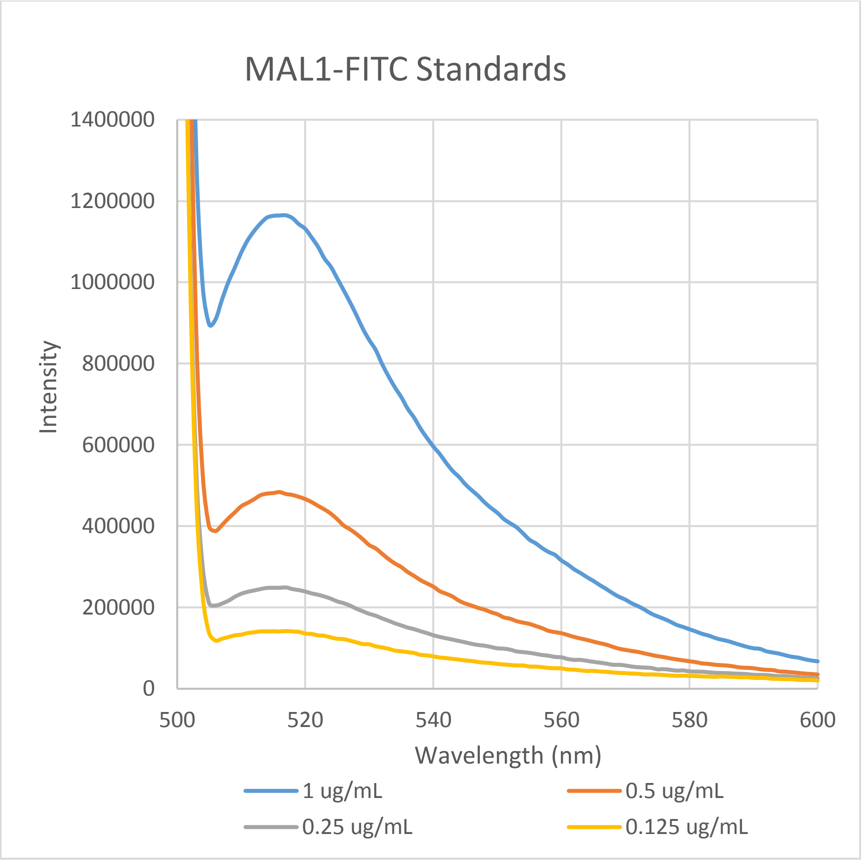

Figure S4. Emission spectra of MAL 1-FITC standards used to determine the amount of Sialyl Lewis X (SLe^X) conjugated to 100 nm FNDs. The concentration of each standard is displayed below the graph. A standard curve was constructed using the emission maxima for each standard (at 515 nm). Standards were excited at 495 nm.

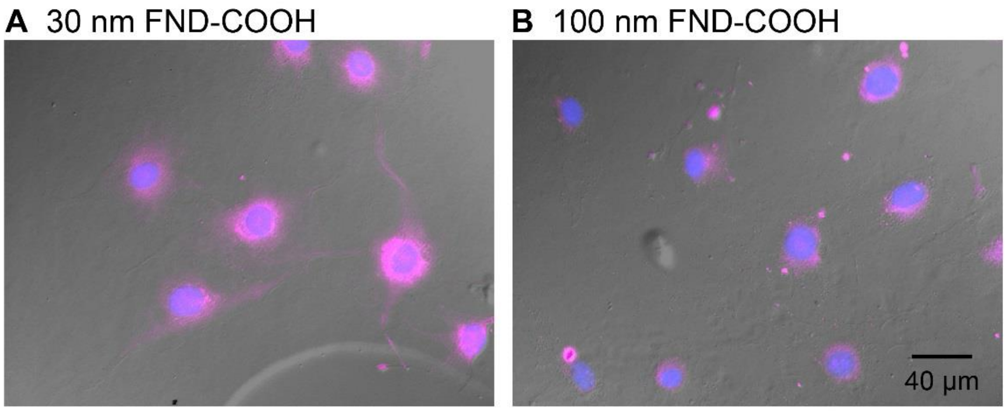

Figure S5. Binding of unmodified FNDs (FND-COOH) to fixed mouse brain endothelial cells. A. 30 nm FND-COOH. **B.** 100 nm FND-COOH. The overlay of DAPI (blue) and FND channels (pink) on the bright field image is shown. The images were acquired with the same exposure time of 14 s on the FND channel. The average brightness of cells containing 30 nm FND-COOH was 32.7 ± 11.2 AU, and the average brightness of cells containing 100 nm FND-COOH was 17.9 ± 7.5 AU.
